# Valorization of Eucalyptus, Giant Reed Arundo, Fiber Sorghum, and Sugarcane Bagasse via Fast Pyrolysis and Subsequent Bio-Oil Gasification

**DOI:** 10.1021/acs.energyfuels.2c01968

**Published:** 2022-09-08

**Authors:** Elmeri Pienihäkkinen, Evert J. Leijenhorst, William Wolters, Christian Lindfors, Joona Lahtinen, Taina Ohra-aho, Anja Oasmaa

**Affiliations:** † VTT Technical Research Center of Finland Ltd., P.O. Box 1000, FI-02044 VTT Espoo, Finland; ‡ BTG Biomass Technology Group B.V., P.O. Box 835, 7500 AV Enschede, The Netherlands

## Abstract

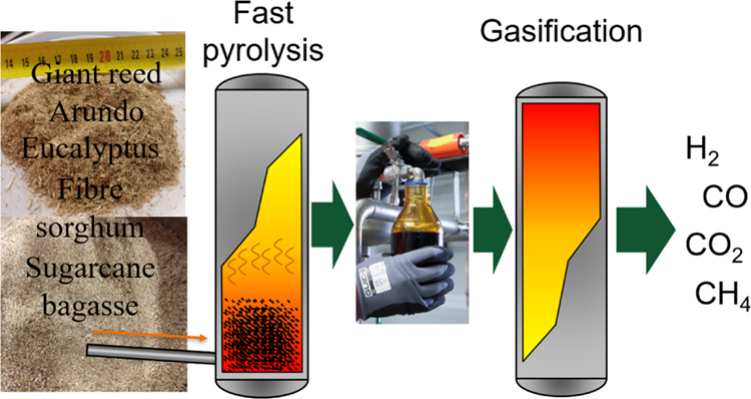

Fast pyrolysis of giant reed Arundo (*Arundo donax*), fiber sorghum (*Sorghum bicolor L.*Moench), eucalyptus (*Eucalyptus* spp.), and sugarcane bagasse (*Saccharum officinarum*) was studied in bench-scale bubbling fluidized bed reactor. Product yields were determined, and detailed physicochemical characterization for produced fast pyrolysis bio-oils (FPBOs) was carried out. The highest organic liquid yield (dry basis) was observed with sugarcane bagasse (59–62 wt %), followed by eucalyptus (49–53 wt %), giant reed Arundo (39 wt %), and fiber sorghum (34–42 wt %). After the pyrolysis experiments, produced FPBOs were gasified in an oxygen-blown autothermal catalytic reforming system for the produced synthesis gas. The gasifier consists of a partial oxidation zone where the FPBO is gasified, and the raw syngas is then reformed over a fixed bed steam-reforming catalyst in the reforming zone. The gas production (∼1.7 Nm^3^/kg FPBO) and composition (H_2_ ∼ 50 vol %, CO 20–25 vol %, and CO_2_ 25–30 vol %) were similar for all FPBOs tested. These results show that the combination of fast pyrolysis with subsequent gasification provides a technically feasible and feedstock flexible solution for the production of synthesis gas.

## 1 Introduction

Production of liquid intermediates by fast pyrolysis in decentralized units and further upgrading of the liquids in centralized upgrading plant has been recognized as a potential strategy to overcome the issues related to the logistics of low-energy-density biomass feedstocks.^1−6^ The liquid produced by fast pyrolysis, i.e., fast pyrolysis bio-oils (FPBOs), has a higher energy density, and they are easier to store and transport to centralized utilization sites compared to bulky biomass feedstocks. In recent years, FPBO production entered the market with currently four pyrolysis plants in operation in Europe.^7^ The primary utilization of the FPBOs is for energy generation through combustion.^8,9^ Recently, Pyrocell started to co-feed FPBO in a fluid catalytic cracking (FCC) system for the production of advanced biofuels as well.^7^ Less mature valorization routes for FPBOs upgrading include hydroprocessing,^10−13^ replacements of fossil resources with FPBO derived fractions for various consumer products,^14^ and gasification of FPBOs into syngas,^15−17^ which was the focus of this study. Compared to direct biomass gasification, the gasification of FPBO has an additional benefit that a large part of the tar precursors (primarily lignin^18^) does not enter the gasifier as it is converted to char in the pyrolysis process. Tar concentrations FPBO gasification are therefore typically much lower than for direct biomass gasification.^19^

Unlike in the case of fast pyrolysis, where the FPBO utilization can be decoupled from the actual production process, gasification cannot be decoupled from the syngas utilization. Syngas can be used as an energy source, or it can be converted into a wide variety of products such as methanol, gasoline, or FT diesel, but the actual syngas utilization must be done at the same site. The economies of scale favor the large plant size, but the size of the plant is typically restricted by the quantity of the economically available biomass resources.^15^ Decentralized production of FPBO could increase the economically available biomass resources and give freedom to build larger gasification plants.^20,21^

As the plant size increases, it is likely that there will be more variation in the type of available biomass feedstocks and FPBOs produced from them. These feedstocks could be blended already in the pyrolysis phase, or the produced FPBOs could be blended in centralized utilization sites.^2,22,23^ In all scenarios, a deep understanding of yields and compositions of the produced FPBOs from different feedstocks are important factors to help in the design of plants, which are capable of handling different feedstocks and product liquids. Several researchers published results on the gasification of pyrolysis oil in various gasifiers, including noncatalytic entrained flow systems^16,24−27^ and various catalytic gasification systems.^17,28−30^ Most research involved the gasification of wood-derived pyrolysis oils, with some straw-derived results included as well. However, a direct comparison of multiple feedstocks in the same system is not previously reported.

In this work, which was part of the Becool project, a cooperation between Europe and Brazil for the production of advanced biofuels,^31^ eucalyptus, giant reed Arundo (later referred to only as Arundo), fiber sorghum (later referred to only as sorghum), and sugarcane bagasse (later referred only as bagasse) were pyrolyzed in bench-scale bubbling fluidized bed reactor. Pyrolysis of sorghum has been studied mainly with small-scale fixed bed,^32^ fluidized bed,^33^ or auger-type reactor systems,^34^ where reported organic liquid yields have been rather low (15–22%). Data produced with larger systems do not exist. The same applies for the Arundo, which has been extensively studied with respect to bio-char production.^35−38^ Data from fast pyrolysis of Arundo are very scarce that is done mainly with small batch-type reactor systems,^39−43^ and reported organic liquid yields from thermal pyrolysis have been low (20 wt %).^41^ On the other hand, data from the fast pyrolysis of bagasse and eucalyptus are more abundant, and typical organic liquid yields from these feedstocks are higher (28–50 wt %).^44−50^ Eucalyptus typically produces FPBO with quality comparable to the other wood feedstock, such as commonly used pine residues.^44,48^ On the other hand, FPBO quality from the bagasse has been reported to vary significantly,^45^ which can be explained by high variability in bagasse feedstock quality.^45,51^

The first goal of this work was to clarify the observable differences in produced fast pyrolysis bio-oils by determining the product yields and by carrying out a detailed physicochemical characterization for produced FPBOs. Further goals were to prove the technical feasibility and assess the efficiency of the FPBO gasification process with varying feedstocks. Related to this work, another publication where a detailed evaluation of the full value chain, from feedstock sourcing up to advanced biofuel production (FT diesel for aviation), is under preparation, and these results will be published later in the literature after completion of the Becool project (2022).

## 2 Materials and Methods

### 2.1 Feedstock Analyses

Feedstocks used for fast pyrolysis tests were Arundo (*Arundo donax*), sorghum (*Sorghum bicolor* L. Moench), and eucalyptus (*Eucalyptus* spp.), which originated from Italy, and bagasse (*Saccharum officinarum*) from Brazil. Arundo, eucalyptus, and first batch of sorghum were dust as received, which was not optimal for pyrolysis. Dust was pelletized and then crushed and sieved to 0.5–1 mm particle size to provide stable feeding. Bagasse and the second batch of sorghum were received as canes, after which they were crushed and sieved at VTT to the desired particle size (0.5–1 mm). Fuel analyses for ground and sieved feedstocks used in the pyrolysis are presented in Table 1.

**Table 1:** Properties for Feedstocks Ground and Sieved to 0.5–1 mm Particle Size^a^

	unit	method	Arundo	eucalyptus	sorghum batch 1	sorghum batch 2	bagasse
moisture	wt %	SFS-EN ISO 18134-3	6.4	6.4	6.2	6.7	7.4
volatiles, dry basis	wt %	SFS-EN ISO 18123	76.3	80.6	77.3	n.m.	83.7
ash 550 °C, dry basis	wt %	SFS-EN ISO 18122	4.4	1.6	5.3	5.8	2.2
carbon, dry basis	wt %	SFS-EN 15104	47.8	49.5	45.5	46.4	48.4
hydrogen, dry basis	wt %	SFS-EN 15104	5.6	5.8	5.7	5.6	5.8
nitrogen, dry basis	wt %	SFS-EN 15104	0.3	0.2	0.8	0.7	0.2
chlorine, dry basis	wt %	SFS-EN ISO16994	0.292	0.121	0.153	n.m.	0.023
sulfur, dry basis	wt %	SFS-EN ISO16994	0.0576	0.0237	0.0619	n.m.	0.031
oxygen, dry basis	wt %	by difference	42	43	42	42	43
HHV, dry basis	MJ/kg	SFS-EN 14918	19.07	19.69	18.15	18.16	19.19
LHV, dry basis	MJ/kg	SFS-EN 14918	17.84	18.43	16.90	16.93	17.92

a n.m. = not measured.

### 2.2 Pyrolysis Experiments

**Figure 1:**
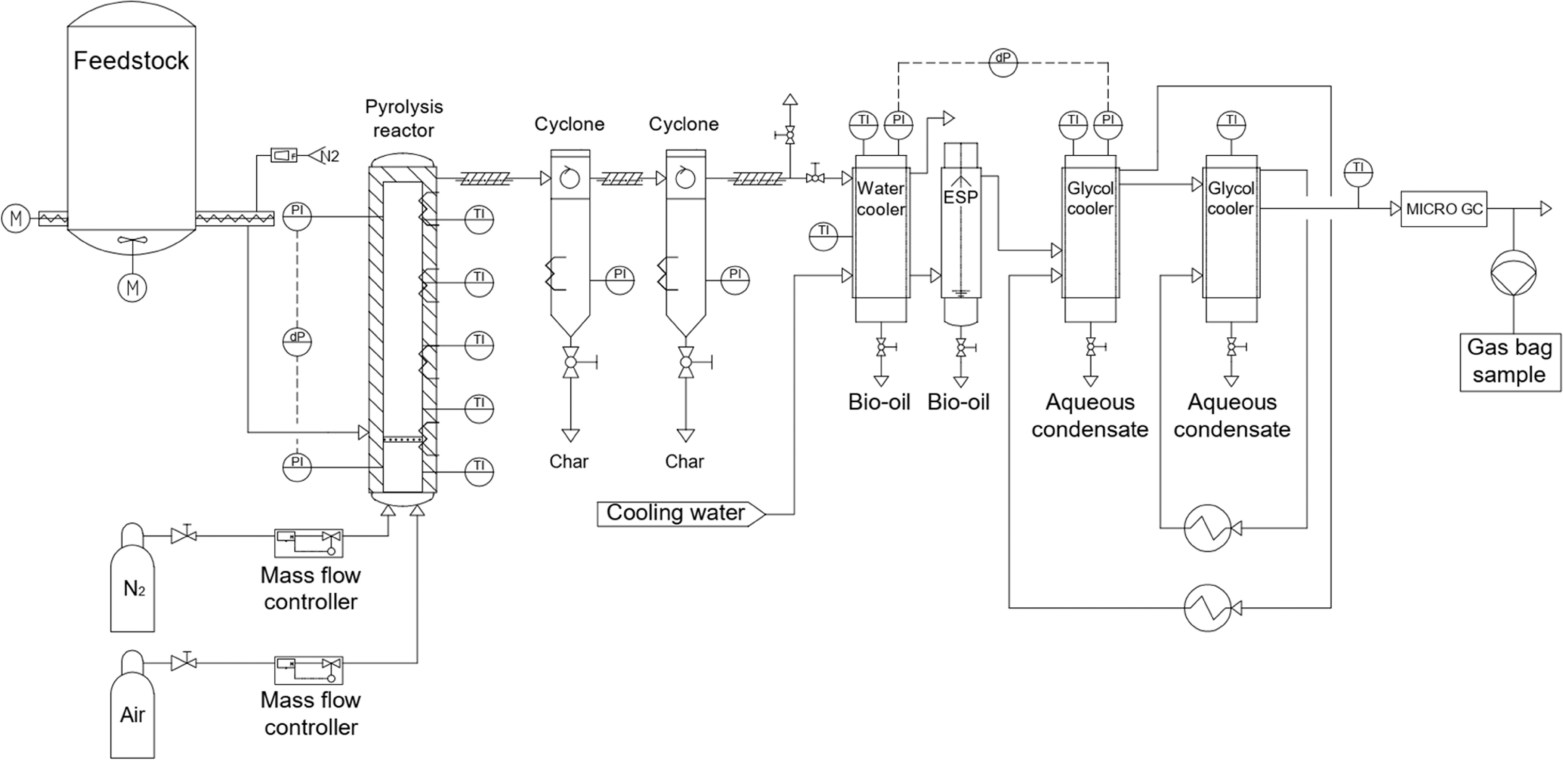
Schematic representation of the bubbling fluidized bed pyrolysis system.

The pyrolysis experiments were performed in a bench-scale bubbling fluidized bed unit (Figure 1). The reactor diameter (*d*) is 52 mm, the height (*h*) is 570 mm, and heat for the pyrolysis is provided from outside with four different electrical heating elements, which enable temperature control through the whole reactor length. The reactor was operated at atmospheric pressure and temperature of 480 °C. The temperature varied ±5 °C during the experiments. In the reactor, 300 g of white aluminum oxide (0.56–0.71 mm, ρ = 4000 g/dm^3^) was used as bed material, which was fluidized by nitrogen. The fluidization gas flow rate was adjusted so that the superficial gas-phase residence time in the reactor conditions was 1 s. The actual residence time during processing was shorter due to the evolution of gases and vapors from the feedstock.

The char was separated from the pyrolysis gases by cyclones. After the cyclones, hot vapors and gases were first cooled indirectly in cold water-cooled heat exchanger (40 °C), after which vapors and gases were passed through an electrostatic precipitator (20 °C) where aerosols from the gases were recovered. From the electrostatic precipitator (ESP) the noncondensed water and light organics were led to two glycol coolers (−10 °C); one-tube heat exchanger and a second smaller-tube heat exchanger filled with additional glass packings. The composition of the noncondensable gases was analyzed by a micro-GC.

After the pyrolysis experiments, the collected char and FPBO were weighed. Most of the organics (≈95 wt %) were recovered in the water-cooled heat exchanger and electrostatic precipitator. The aqueous condensate recovered in the glycol cooler contained 80–85 wt % of water. The liquid products recovered in the water-cooled heat exchanger and electrostatic precipitator were weighed and mixed before physicochemical characterization. The liquid recovered from the glycol coolers was weighed and treated separately. The liquid recovery system was rinsed after each experiment with a small amount of methanol to remove the condensed bio-oils from the walls of the condensers. The amount of pyrolysis liquid condensed on the walls was determined by evaporating the methanol from the washing liquid with a rotavapor, weighing the residue, and analyzing its water content. The total FPBO yield was the sum of all of these different recovered liquid fractions. Product yields are reported on a dry basis from the starting feedstock. Organic liquid yield refers to dry organics in the liquid fractions. Pyrolytic water refers to water formed in the pyrolysis reactions (pyrolytic water = total water in liquid products – moisture of the feedstock).

### 2.3 Pyrolysis Product Characterization

Physical characterization of the liquids was carried out by employing modified standard methods.^52^ Water content was analyzed by Karl Fischer titration using a Metrohm 795 KFT Titrino titrator (ASTM E 203). Elemental composition analysis (CHN) was carried out using an Elementar VARIOMAX CHN analyzer (ASTM D 5291), and a higher heating value (HHV) was determined using an IKA Werke C 5000 Control calorimeter (DIN 51900). Carboxylic acid number (CAN) was determined with a 785 DMP Titrino analyzer (ASTM D 664), and micro carbon residue (MCR) was analyzed using an Alcor Micro Carbon Residue Tester (ASTM D 4530). The ash content of the liquid was further determined by combusting the residue from the MCR determination in a muffle furnace at 775 °C. The viscosity of the liquid was measured with a Cannon-Fenske viscometer (ASTM D 445), and the density of the liquid was measured with an Anton Paar DMA 4500M analyzer (ASTM D 4052). Carbonyls were measured by titration with the method described elsewhere.^53^ FPBO stability was measured by keeping the oil at 80 °C for 24 h and measuring the change in viscosity, water, and carbonyl content.^52^ The homogeneity of liquid was determined by optical microscopy.

The chemical composition of the FPBOs was determined with the solvent fractionation scheme. In this method, FPBO is first divided into a water-soluble (WS) and a water-insoluble (WIS) fraction by water extraction. The water-soluble fraction is further extracted with diethyl ether to an ether-soluble (ES) and an ether-insoluble (EIS, sugar-like material) fraction. The water-insoluble fraction is extracted with dichloromethane (DCM) to a DCM-soluble fraction containing low-molecular-mass (LMM) lignin and a DCM-insoluble fraction containing high-molecular-mass (HMM) lignin. In general, the LMM fraction contains poorly water-soluble lignin monomers and dimers (MM = 400 Da) and extractives, while the HMM fraction contains powder-like high-molecular-mass (MM = 1050 Da) lignin-derived material and solids.^52,54^

### 2.4 Gasification Experiments

**Figure 2:**
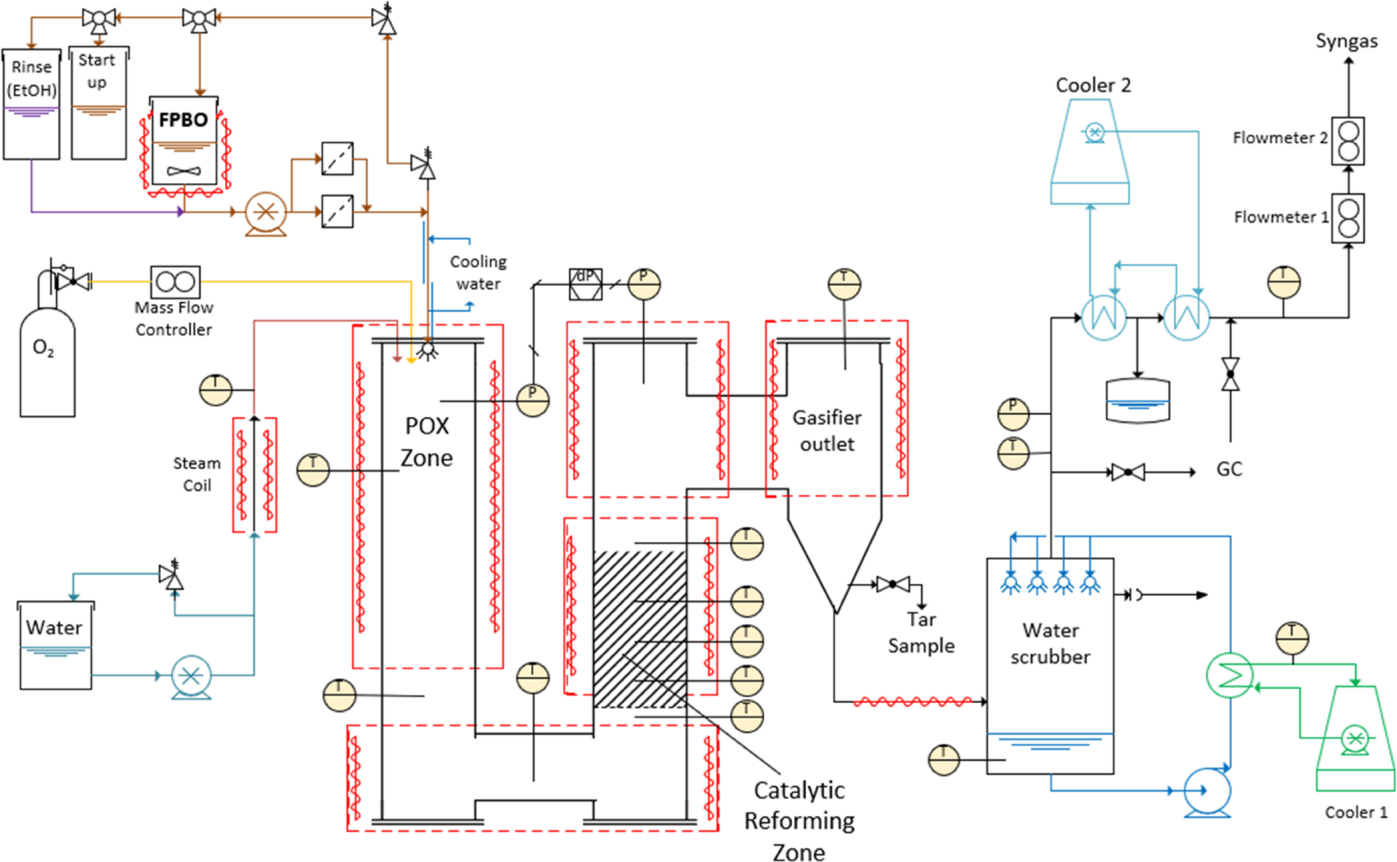
Schematic representation of the autothermal catalytic reforming system.

Gasification of the FPBO for syngas production was performed in a bench-scale system (Figure 2) converting about 3 kg FPBO/h. Before gasification, 20 wt % bioethanol (99% pure, bioethanol shop) was mixed into the FPBO to reduce the viscosity and improve atomization in the bench-scale setup. The influence of ethanol on the gasification performance was determined before the current set of experiments using variable ratios of ethanol and wood-derived FBPO. Ethanol is found to slightly increase the energy efficiency as well as the hydrogen content in the syngas, which can be explained by the higher heating value and hydrogen content of ethanol compared to FPBO. For larger-scale gasifier systems mixing ethanol is not required for processing and pure FPBO can be used.^16,25^ The gasifier consists of two directly connected reactor zones. In the partial oxidation zone (POX zone), FPBO is atomized and mixed with oxygen and steam to form raw syngas, and in the fixed bed catalytic reforming zone, the hydrocarbons present in the raw syngas (tars, but also smaller hydrocarbons such as methane, ethene, etc.) are reformed to produce a clean syngas.

FPBO is supplied to the gasifier at a high pressure using a piston pump (Williams, CRP750V400). Atomization in the gasifier (operated at atmospheric pressure) is achieved using a spray nozzle (Spraybest, HA-63-G15). This spray nozzle consists of a very small hole (0.15 mm) through which the FPBO is pumped, resulting in good atomization. The hole size determines the feed capacity, together with the pressure difference. Initial tests with a smaller system (0.10 mm) showed rapid clogging of the nozzle, while tests with a larger hole (0.20 mm) increased the capacity beyond that acceptable for the rest of the system. For large-scale gasifiers, a larger capacity is acceptable and atomization will be less of an operational issue.

The atomized FPBO is mixed with pure oxygen (Brooks MFC) from a gas bottle (Linde, 99.9%) and steam produced from demineralized water pumped (Williams CRP250V300) through a heated coil. The gasifier is preheated to the desired operating temperature (here 900 °C) using an electrical oven (Westeneng ovenbouw BV). The catalyst used in the reforming zone is a commercially available catalyst (ReforMax 330 LDP with nickel as the active component) purchased from Clariant. The hot syngas is cooled after the gasifier using a water scrubber and several indirect heat exchanges to further cool the gas to room temperature before analysis. The gasifier is monitored and controlled using a National Instruments data acquisition system combined with Labview Software. Liquid feed capacities were measured using Sartorius scales, and gas production is measured using an ultrasonic volume flow meter (Krohne Optisonic 7300). Water production was measured by weighing the water content in the scrubber before and after each experiment. Finally, the gas composition was measured using a Synspec GC-955 system able to determine the H_2_, O_2_, N_2_, CO, CO_2_, CH_4_, C_2_H_4_, C_2_H_6_, C_3_H_6_, and C_3_H_8_ concentrations.

The experiments were performed by preheating the gasifier to 900 °C, using a target FPBO feed capacity of 2.5 kg/h, with 1.25 kg/h oxygen and 2.4 kg/h steam. The actual feed capacity is achieved depending on the FPBO pressure and viscosity of the liquid. The oxygen and steam amounts were slightly adjusted if needed to control the temperature in the gasifier (900 °C in the partial oxidation zone, 850 °C in the catalytic reforming zone) before the steady-state measurement period started (settings in Table 2). The eucalyptus and sorghum-derived FPBO resulted in higher FPBO capacities, lowering the actual fuel:oxygen and fuel:steam ratios slightly.

**Table 2:** Operating Conditions of the Gasification

	unit	Arundo	eucalyptus	sorghum	bagasse
FPBO in	kg/h	2.4	2.9	3.2	2.7
oxygen in	kg/h	1.2	1.2	1.3	1.3
steam in	kg/h	2.6	2.4	2.7	2.6

The cold gas efficiency in the gasifier is calculated by dividing the product of the LHV and mass flow of the cold product gas with the product of LHV and mass flow of the pyrolysis oil. During gasification, the electrical ovens, which are used to preheat the system, are switched on; however, the operator ensures that the process conditions generated within the gasifier are at a higher temperature than the ovens.

## 3 Results and Discussion

### 3.1 Pyrolysis Experiments

Fast pyrolysis experiments were carried out with eucalyptus, Arundo, sorghum, and bagasse. Product yields from the successful experiments are presented in Table 3. In the case of sorghum, two separate batches of FPBO were produced, one from each feedstock batch received. Oil batch numbers correspond to the similarly named feedstock batches.

**Table 3:** Product Yields Calculated on Dry Basis for the Bench-Scale Fast Pyrolysis Experiments at 480 °C

feedstock batch	Arundo	eucalyptus		sorghum batch 1		sorghum batch 2		bagasse	
duration, h	5.9	8.0	9.3	4.0	4.1	6.0	8.0	6.1	3.0
feed rate, g/h	1531	702	1226	854	558	722	747	1165	1256
mass balance, wt % on dry mass basis									
char	33	21	20	24	24	26	27	16	17
gases	9	12	15	13	14	20	19	12	10
organic liquid	39	53	49	42	34	42	41	62	59
pyrolytic water	15	10	12	16	12	11	10	9	9
Sum	96	97	95	95	84	99	97	99	96

**Figure 3:**
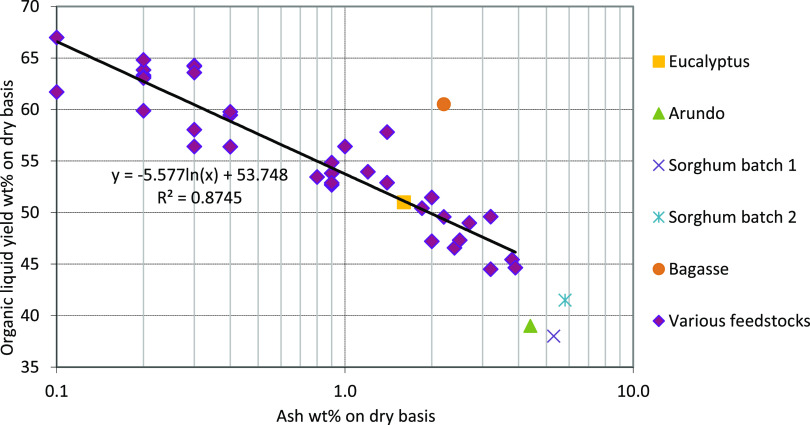
Organic liquid yields as a function of the ash content in addition to data from experiments carried out with various feedstock with different ash contents in the VTT’s fluidized bed pyrolysis units.^48^

When the product yields are considered, the organic liquid yields from bagasse runs were the highest (59–62 wt %). In addition, the char, gas, and pyrolytic water yields were the lowest with bagasse. With eucalyptus, decent organic liquid yield was also achieved (49–53 wt %), but with Arundo and sorghum, the yields were rather low (39 and 34–42 wt %, respectively). Bagasse and eucalyptus have lower ash contents but also higher volatile contents, which are expected to be the main reasons for higher organic liquid yields.^55^ Organic liquid yields as a function of ash content are presented in Figure 3. Most of the feedstock ash is expected to end up in the char,^56^ but the ash content of the char was not analyzed in this study. The yield of bagasse was significantly higher than could be expected only from the feedstock ash content. Although ash content generally correlates rather well with the yield, it has been reported that the alkali and alkaline earth metals (AAEM) within the ash are the most important elements affecting the FPBO yield.^48^ Feedstock AAEM content is expected to be the explanation for observed yields but a more detailed analysis of the feedstock ash content would be needed to confirm this. Volatile content of the feedstock correlated also rather well with the organic liquid yield (Table 1) as the organic liquid yield was higher with increasing volatile content of the feedstock. Bagasse had the highest volatile content (83.7 wt %), followed by eucalyptus (80.6 wt %), sorghum (77.3 wt %), and Arundo (76.3 wt %). Yield from eucalyptus was in a similar range to that reported in other studies using fluidized bed reactors.^44,48^ Although the yield of bagasse was high, similar FPBO yields (organics + water) have been reported previously. Montoya et al.^57^ reported an FPBO yield of 70.9 wt % from bagasse in the BFB reactor which is in a similar range as achieved in this study (71 wt %). However, the water content of the FPBO was not reported by Montoya et al.

Regarding the operational observations, there were no problems in the experiments with eucalyptus and bagasse. With Arundo and sorghum, problems with ESP were observed. In the case of sorghum, problems were severe and led to premature termination of the experiments and prevented the long operational periods. Current struck through the electrode of ESP and led to the melting of Teflon parts of the device. To solve the issue, a nitrogen purge was installed at the top of the ESP with the aim to keep the top part of the ESP cleaner and prevent operational problems. This was found to be the practical solution for the problems, and after this modification, longer stable operational periods were successful. The reason why these problems were severe only with sorghum remained unclear.

Regarding the feedstock composition, sorghum has the highest content of nitrogen (0.7–0.8 wt %, Table 1), which contributes to the high nitrogen content of sorghum-derived FPBO (0.9–1.0 wt %, Table 4). Based on the nitrogen content, a rough estimate of protein content of feedstocks can be carried out by multiplying the dry nitrogen content by a factor of 6.25.^58^ This gives protein contents of 1.9, 1.3, 5.0, 4.4, and 1.3 wt % on a dry basis for Arundo, eucalyptus, sorghum batch 1, sorghum batch 2, and bagasse, respectively. Pyrolysis products of proteins and amino acids include many basic nitrogen compounds such as ammonia and amines,^59^ which can be a reason for the higher pH (3.5) of sorghum FPBO compared to the other FPBOs (2.3–2.9). Basic nitrogen components can also add free ions into the FPBOs, which increase the electrical conductivity and can explain the problems observed at the ESP. Regarding the carboxylic acid (CAN) and carbonyl content of the FPBOs from eucalyptus, Arundo, and sorghum, there were no major differences. However, the bagasse FPBO had slightly lower pH, higher CAN, and higher carbonyls compared to the other FPBOs. In addition, bagasse FPBO was higher in density and viscosity, which can be explained by lower water content, and was more stable with respect to viscosity changes during the stability test.

**Table 4:** Physical and Chemical Properties of the FPBOs Analyzed as Received^a^

feedstock	Arundo	eucalyptus	sorghum^b^	sorghum^b^	bagasse	EN 16900:2017
			batch 1	batch 2		Standard, Grade 2
water, wt %	29.6	22.8	26.1	29.5	17.7	<30
solids, wt %	0.25	0.14	0.63	0.7	0.68	<0.5
MCR, wt %	16.6	18.6	17.5	16.4	20.7	
Ash, wt %	0.16	0.03	0.09	0.08	<0.01	≤0.05^c^
Na + K, wt %						≤0.02^c^
carbon, wt %	41.2	43.0	41.4	41.3	43.9	
hydrogen, wt %	8.0	7.7	8.0	8.2	7.1	
nitrogen, wt %	0.3	0.2	0.9	1.0	0.2	
chloride, mg/kg	260	240	133	76	130	
sulfur, mg/kg	338	144	528	525	139	≤0.05^c^
oxygen (as diff.), wt %	50	49	50	49	49	
viscosity (20 °C), cSt	34	66	37	n.m.	153	
viscosity (40 °C), cSt	11	20	13	14.2	42	≤50
viscosity (60 °C), cSt	5.1	7.1	5.8	n.m.	11.8	
viscosity (80 °C), cSt	2.7	3.8	3.1	n.m.	5.1	
density (20 °C), kg/dm^3^	1.162	1.191	1.171	n.m.	1.229	≤1.3^d^
density (40 °C), kg/dm^3^	1.145	1.174	1.154	1.140	1.213	
density (60 °C), kg/dm^3^	1.126	1.156	1.124	n.m.	1.187	
density (80 °C), kg/dm^3^	1.106	1.135	1.113	n.m.	1.174	
pour point, °C						≤− 9
HHV, MJ/kg	17.25	17.89	17.63	17.58	17.94	
LHV, MJ/kg	15.50	16.21	15.88	15.80	16.39	≥14
pH	2.9	2.8	3.5	3.4	2.3	≥2
CAN, mg KOH/g	88	82	83	89.5	116	
carbonyls, mmol/g	3.7	4.2	4.2	3.2	5.6	
stability test 24 h 80 °C						
water increase, %	2	9	n.a.	n.a.	10	
viscosity increase, %	143	94	n.a.	n.a.	52	
carbonyl decrease, %	32	36	n.a.	n.a.	36	
after stability test						
water, wt %	30.2	24.9	n.a.	n.a.	19.4	
viscosity (40 °C), cSt	28	39	n.a.	n.a.	64	
carbonyls, mmol/g	2.5	2.7	n.a.	n.a.	3.6	

a n.a. = not applicable, heterogeneous product after stabilization.

b Sorghum FPBO from batch 1 and batch 2 were mixed before gasification.

c On dry basis.

d Measured at 15 °C.

On the other hand, sorghum FPBO was not stable during the stability test. A heterogeneous product was obtained after the stabilization test, and reliable analysis results were not achieved. A solid layer was formed on top of the oil phase. Agro-biomasses contain proteins consisting of long chains of amino acid residues, which can be decomposed into several types of nitrogen-containing compounds, the detailed structure and solubility of which are unknown. Another reason for the top phase can be extractives; however, due to the small quantity, the solid layer could not be characterized.

**Figure 4:**
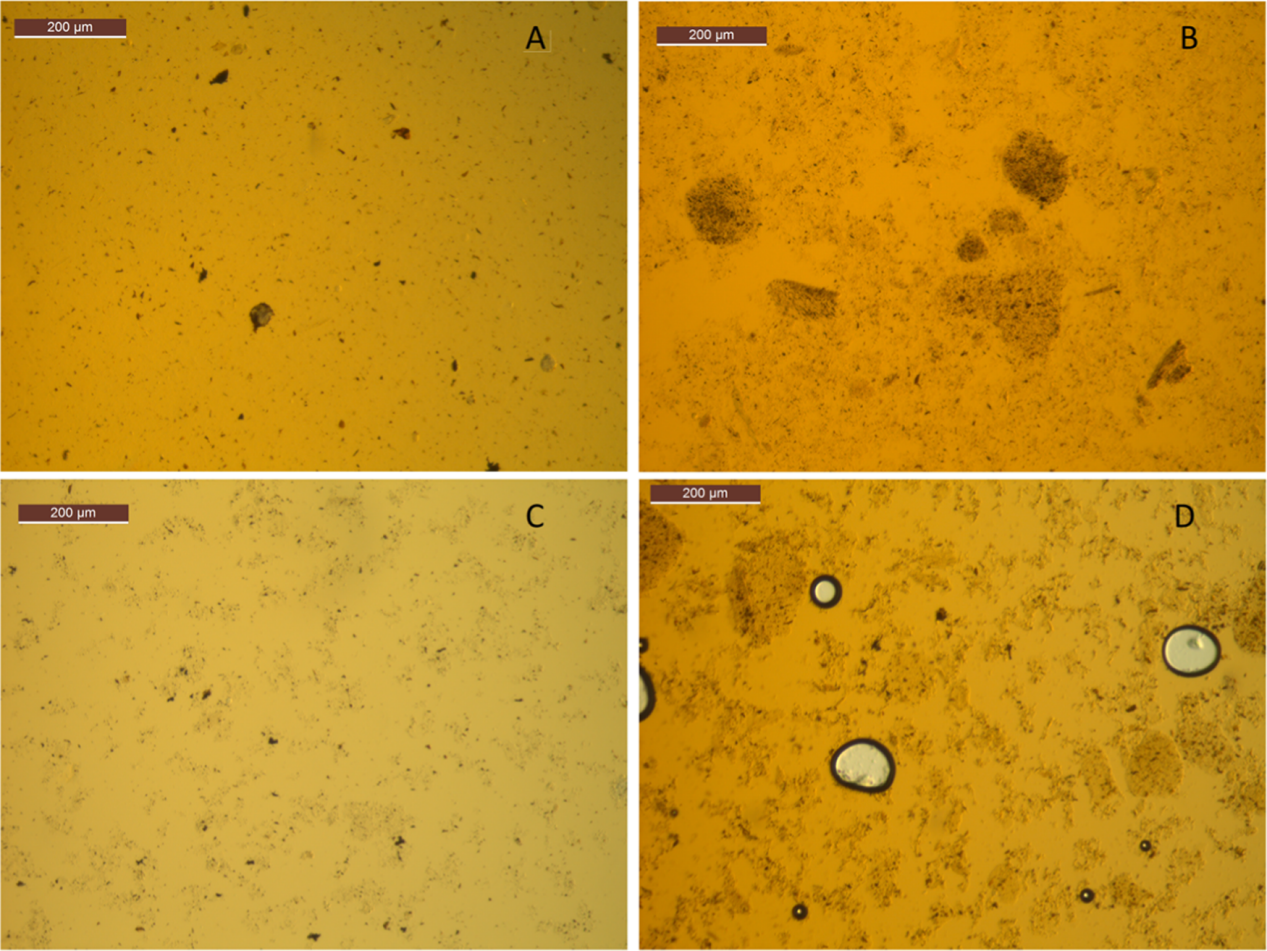
Microscopic images of the FPBOs: (A) Bagasse, (B) Arundo, (C) eucalyptus, and (D) sorghum batch 1. Black dots are solid particles. Bubbles with black surroundings are air bubbles.

**Figure 5:**
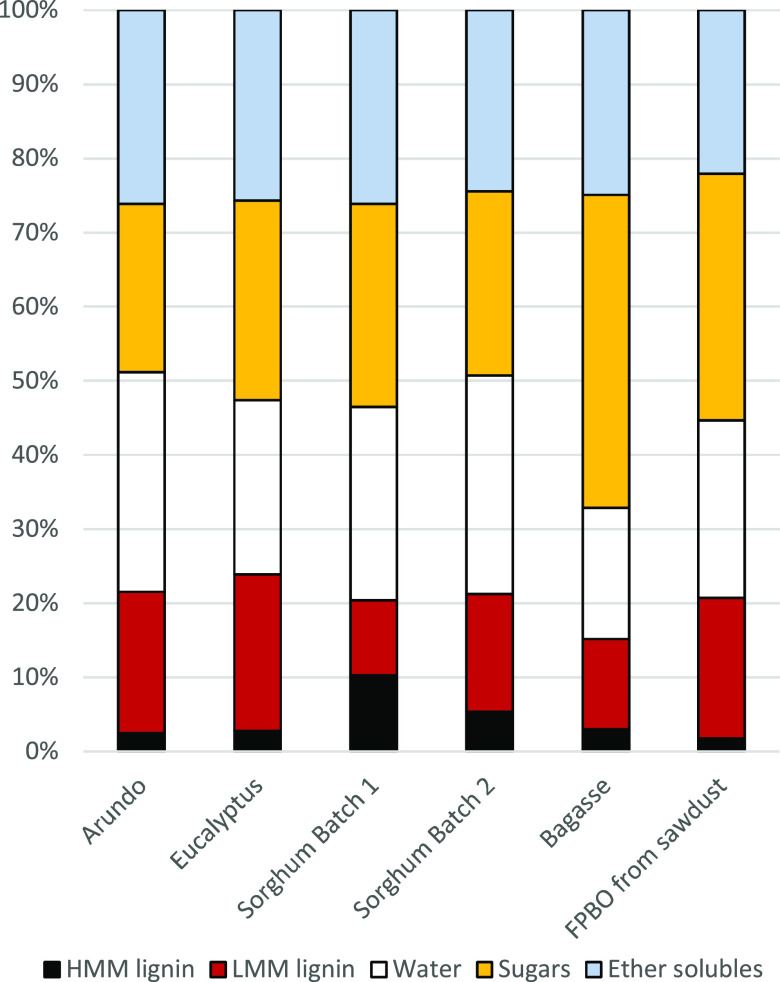
Chemical composition of produced FPBOs compared with FPBO from pine sawdust. Data from sawdust FPBO were acquired from Oasmaa et al.^55^

Microscopic images of the FPBOs from eucalyptus, Arundo, bagasse, and sorghum batch 1 are presented in Figure 4, and the chemical composition of all FPBOs is shown in Figure 5. All FPBOs were visually homogeneous products even if the water content with Arundo was 29.6 wt %. A clear difference in the chemical composition of the FPBOs is the high HMM (high-molecular-mass) fraction in sorghum FPBOs. This was especially high in sorghum Batch 1. Otherwise, the composition of sorghum FPBOs was similar. The slightly higher solids content of sorghum FPBOs only partly explains the higher HMM content. Also, extractives typically can be found in the LMM fraction and not in HMM.^54^ The higher HMM could be due to some nitrogen-containing compounds which are insoluble in water and dichloromethane, as there should not be a major difference in the lignin content feedstocks. Another clear difference is the higher sugar and lower lignin contents in bagasse FPBO compared to other oils.

If the quality of the liquids is considered with respect to standard EN 16900–2017, which specifies the FPBO quality for industrial boiler use, the sulfur content of sorghum FPBOs, the solids contents of sorghum and bagasse FPBOs, and the ash content of Arundo and sorghum FPBO exceed the limit values specified for Grade 2 FPBO^60^ (Table 4) Otherwise, all of the criteria for FPBOs in industrial boiler use (>1 MW thermal capacity) are fulfilled. Centralized gasification of different quality FPBOs could be a robust and viable option for FPBO upgrading, and the technical feasibility of the gasification for the produced FPBOs is assessed in the following section.

### 3.2 FPBO Gasification

To ensure proper atomization of the FPBOs in the gasifier, 20 wt % bioethanol was added to reduce the viscosity of the fuel and prevent the formation of large droplets. This requirement is particularly important for the small scale of the experimental setup; for a full-scale gasifier, pure FPBO can be used as discussed in the Introduction section. The composition of the fuels as fed to the gasifier is presented in Table 5. Here, the FPBOs are named according to the original biomass feedstock with the bioethanol content added as a subscript for clarity. For sorghum, the FPBOs from batch 1 and batch 2 were combined before mixing with bioethanol.

**Table 5:** Composition of the FPBOs as Fed to the Gasifier, All Data on “As Received” Basis

	unit	Arundo_20_	eucalyptus_20_	sorghum_20_^a^	bagasse_20_
ethanol added	wt %	20	20	20	20
carbon	wt %	43.4	45.8	42.9	44.8
hydrogen	wt %	8.9	8.5	8.4	8.0
nitrogen	wt %	0.4	0.3	0.7	0.2
oxygen (by diff)	wt %	47.3	45.4	48.0	46.9
water	wt %	22.9	19.8	25.6	14.8
LHV	MJ/kg	18.0	18.7	17.1	17.9
viscosity (40 °C)	cSt	7	12	8	18
MCR	wt %	13	15	13	16

a Sorghum oil from batch 1 and batch 2 were combined for the gasification tests.

For the Arundo and eucalyptus-derived FPBOs, a 2 h run could be performed without operational problems. Evaluation of the data showed that steady state was achieved rapidly with very stable gas compositions throughout the tests. The first tests with sorghum-derived FPBO resulted in an emergency shutdown after about 0.5 h caused by a bad thermocouple connection. This incident was unrelated to the sorghum FPBO or the experiment as such. A second test with sorghum-derived FPBO was started a few days later after cleaning the system and ran for around 1 h before all fuel was consumed. Only results from the second run with sorghum are used in the evaluation; however, the gas composition in the first test was similar. For the bagasse-derived FPBO, an initial run of about 1 h was performed, after which the system was manually shut down to evaluate the results and save some FPBO for a second test and test reproducibility with this feedstock. During the second test, the oxygen flow to the gasifier needed to be decreased (from 1.3 to 1.1 kg/h) to prevent the too high temperature in the partial oxidation zone. The high temperatures are previously encountered in other experiments and are caused by bad atomization. When not all FPBO is properly atomized, part of the FPBO does not react sufficiently quickly in the gasifier, which results in a lower apparent fuel:oxygen ratio and, therefore, at a higher temperature. After about 0.5 h, the test was terminated because of the poor atomization. The somewhat higher viscosity of the bagasse-derived FPBO (20 versus 7–12 cSt, see Table 5) is a likely cause of the poor atomization, but this could not be confirmed. Unfortunately, not enough FPBO remained for a third trial. For the bagasse-derived FPBO, only results from the first run were used.

**Figure 6:**
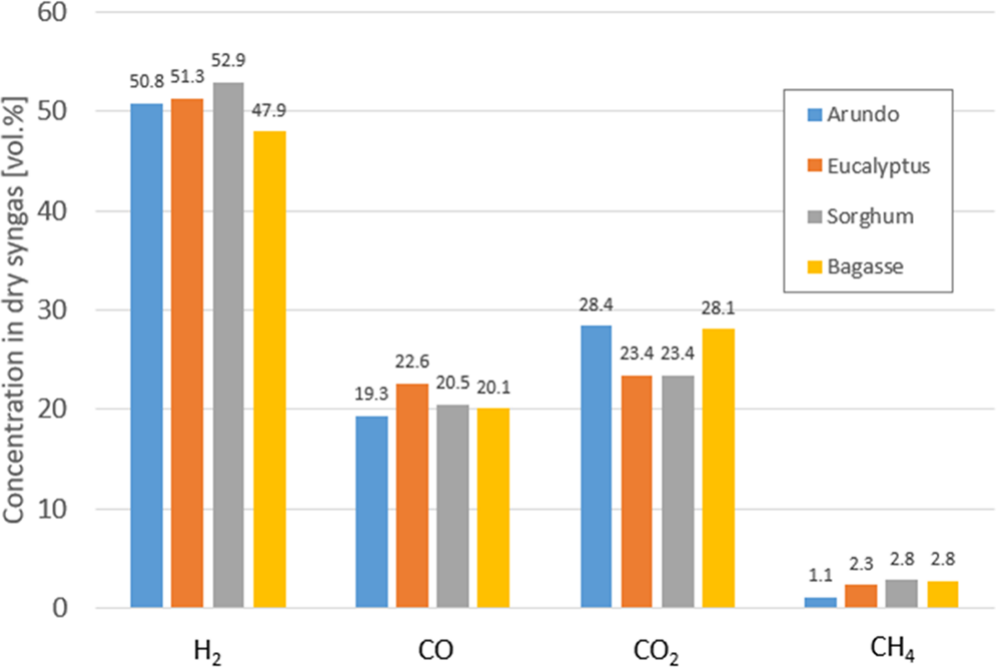
Dry syngas composition obtained with the four FPBOs.

The dry syngas composition for the four FPBOs is presented in Figure 6. Hydrogen was around 50 vol % for all feedstocks, with 19–23% CO and 23–28 vol % CO_2_. The H_2_/CO/CO_2_ concentrations (and that of H_2_O) were close to the thermodynamic equilibrium of the water–gas shift reaction. The total syngas production was 1.71 Nm^3^/kg FPBO for Arundo, 1.75 Nm^3^/kg FPBO for eucalyptus, 1.68 Nm^3^/kg FPBO for sorghum, and 1.68 Nm^3^/kg FBPO for bagasse, showing that not only the gas composition but also the gas production is similar for all for fuels.

Methane was present in low concentrations (1.1–2.8 vol %) in the syngas, which is above the equilibrium value predicted by the methane-steam-reforming equilibrium. The methane reforming was not complete, which is frequently observed in gasification systems.

The carbon-to-gas ratio depends primarily on the atomization performance, which could not be optimized for these tests but still was considered to be quite good (Arundo 0.98, eucalyptus 0.94, sorghum 0.94, and bagasse 0.98). The cold gas efficiency was 76% for Arundo, 83% for eucalyptus, 89% for sorghum, and 80% for bagasse. Cold gas efficiencies around 80% are close to the theoretical maximum; therefore, the measurement for the sorghum test seems overestimated. In Table 4, the LHV of sorghum FPBO (15.88) without ethanol was between that of Arundo FPBO (15.5) and Eucalyptus (16.2), while after ethanol addition, the LHV for sorghum_20_ was significantly lower than all other oils (Table 5). This is a direct consequence of the relatively low hydrogen content measured for sorghum_20_. In case the LHV from Table 4 is used and corrected for the ethanol blending, the cold gas efficiency of the sorghum tests drops to 83%, which is a much more realistic value. However, to establish a proper data set on the gasification performance, more FPBO would be required to perform duplicate measurements and investigate the influence of process conditions on the performance; however, in this paper, only screening experiments were performed. The LHV for sorghum_20_ reported in Table 5 was significantly lower compared to the LHV of the other fuels, caused primarily by a relatively low carbon content. Unfortunately, the sample was not available anymore for re-analysis.

## 4 Conclusions

Eucalyptus, Arundo, sugarcane bagasse, and two batches of sorghum were pyrolyzed in bench-scale BFB reactor. Product yields were determined, and detailed physicochemical characterization was carried out for produced FPBOs. Eucalyptus, Arundo, and bagasse were successfully pyrolyzed, but with sorghum, severe problems with the liquid recovery in the ESP were observed.

Ash content decreased the organic liquid yield for all feedstocks during pyrolysis, except with the bagasse, which gave a much higher organic liquid yield than could be expected only from the feedstock ash content. All produced liquids were homogeneous after the experiments, but the FPBO from sorghum was not stable and produced heterogeneous products after the stabilization test. A clear difference in chemical compositions of the FPBOs was the high HMM (high-molecular-mass) fraction in sorghum FPBO. The higher HMM content could be due to extractives or some nitrogen-containing compounds which are insoluble in water. The highest organic liquid yield was obtained with bagasse. In addition, bagasse FPBO contained more sugars and less lignin and water and had the lowest pH and highest acid number compared to other produced FPBOs.

Subsequent gasification of the FPBOs resulted in a relatively similar syngas production and composition. Hydrogen was around 50 vol % for all feedstocks, with 19–23% CO and 23–28 vol % CO_2_, and the gas concentrations were close to the thermodynamic equilibrium of the water–gas shift reaction. The total syngas production was 1.71 Nm^3^/kg FPBO for Arundo, 1.75 Nm^3^/kg FPBO for Eucalyptus, 1.68 Nm^3^/kg FPBO for sorghum, and 1.68 Nm^3^/kg FBPO for bagasse, showing not only the gas composition but also the gas production is similar for all for fuels. These results show that the combination of fast pyrolysis and gasification indeed provides a feedstock flexible value chain for the production of advanced biofuels from biomass residues.
